# Examining Microstructural White Matter in Active Duty Soldiers with a History of Mild Traumatic Brain Injury and Traumatic Stress

**DOI:** 10.2174/1874440001711010046

**Published:** 2017-09-06

**Authors:** Michael N. Dretsch, Rael T. Lange, Jeffery S. Katz, Adam Goodman, Thomas A. Daniel, Gopikrishna Deshpande, Thomas S. Denney, Grant L. Iverson, Jennifer L. Robinson

**Affiliations:** 1 US Army Aeromedical Research Laboratory, Fort Rucker, AL; Human Dimension Division, Headquarters Training and Doctrine Command, 950 Jefferson Ave, Fort Eustis, VA, 23612, USA; 2 National Intrepid Center of Excellence, Defense and Veterans Brain Injury Center, Walter Reed National Military Medical Center, Palmer Road, Bethesda, MD, 20814, USA; 3 Department of Psychology, 226 Thach Hall, Auburn University, Auburn, AL, 36849, USA; 4 Auburn University MRI Research Center, Department of Electrical & Computer Engineering, 570 Devall Drive, Auburn University, Auburn, AL, 36832, USA; 5 Alabama Advanced Imaging Consortium, Auburn University and University of Alabama Birmingham, AL, USA; 6 Department of Physical Medicine and Rehabilitation, Spaulding Rehabilitation Hospital, 300 First Avenue, Harvard Medical School, Boston, MA 02129; & Home Base, A Red Sox Foundation and Massachusetts General Hospital Program; and Defense and Veterans Brain Injury Center, Bethesda, MD, USA

**Keywords:** DTI, MRI, Traumatic brain injury, TBI, PTSD, Military

## Abstract

**Background:**

There is a high comorbidity of posttraumatic stress (PTS) and mild traumatic brain injury (mTBI), with largely overlapping symptomatology, in military service members.

**Objective:**

To examine white matter integrity associated with PTS and mTBI as assessed using diffusion tensor imaging (DTI).

**Method:**

Seventy-four active-duty U.S. soldiers with PTS (n = 16) and PTS with co-morbid history of mTBI (PTS/mTBI; n = 28) were compared to a military control group (n = 30). Participants received a battery of neurocognitive and clinical symptom measures. The number of abnormal DTI values was determined (>2 SDs from the mean of the control group) for fractional anisotropy (FA) and mean diffusivity (MD), and then compared between groups. In addition, mean DTI values from white matter tracts falling into three categories were compared between groups: (i) projection tracts: superior, middle, and inferior cerebellar peduncles, pontine crossing tract, and corticospinal tract; (ii) association tracts: superior longitudinal fasciculus; and (iii) commissure tracts: cingulum bundle (cingulum-cingulate gyrus and cingulum-hippocampus), and corpus callosum.

**Results:**

The comorbid PTS/mTBI group had significantly greater traumatic stress, depression, anxiety, and post-concussive symptoms, and they performed worse on neurocognitive testing than those with PTS alone and controls. The groups differed greatly on several clinical variables, but contrary to what we hypothesized, they did not differ greatly on primary and exploratory analytic approaches of hetero-spatial whole brain DTI analyses.

**Conclusion:**

The findings suggest that psychological health conditions rather than pathoanatomical changes may be contributing to symptom presentation in this population.

## 1 INTRODUCTION

A large number of military service members have sustained a traumatic brain injury (TBI) while on active duty, the majority of which are categorized in the mild range (mTBI) [1]. Although the majority of individuals are expected to fully recover following mTBI, a small but significant number continue to report ongoing symptoms many months or years following injury [2]. To complicate matters, there is a high prevalence of posttraumatic stress (PTS) in service members who deployed for Operation Iraqi Freedom, Operation Enduring Freedom, and Operation New Dawn [3-7]. In service members that go on to develop persistent postconcussive symptoms, due to the high overlap of symptoms associated with PTS, teasing apart the etiology of the symptoms can be difficult [6, 8, 9]. There is no effective conventional approach beyond clinician judgment to apportion etiology (mTBI vs. PTS) for persistent symptoms. In the brain, various biochemical changes following neural injury in TBI include altered protein trafficking, protein aggregation, complement activation, altered cytoskeletal organization and other alterations [10, 11]

Patients with mTBI usually do not have macrostructural evidence of brain injury visible on conventional neuroimaging, such as T1- or T2-weighted magnetic resonance imaging (MRI) [12, 13]. However, other techniques, such as diffusion tensor imaging (DTI), have shown promise toward understanding the pathophysiological impact of mTBI [14-16], and more recently, traumatic stress on white matter tracts [16-20].

Fractional anisotropy (FA) and mean diffusivity (MD) are common DTI metrics used to infer white matter integrity [21]. FA infers the directional coherence of water diffusivity (anisotropy) to the anatomical white matter tracts [22]. MD is the average diffusion within a specified voxel or region of interest [23, 24]. MD is thought to be an inverse measure of membrane density in which greater values are associated with axonal degeneration, whereas lower values are associated with high myelination and dense axonal packing [22, 25]. Together, these quantitative measures allow researchers to draw inferences about the integrity of white matter.

There is great variability between mTBIs when considering the mechanism, biomechanics, and localization (*e.g.*, frontal, temporal, posterior, or combination) associated with the neural injury. Methodological differences and variability in injury characteristics can account for differences in results between studies, which might reflect why DTI findings in mTBI remain inconsistent at best [see review, 14]. In an attempt to address the spatial heterogeneity of possible damage to white matter following mTBI, recent approaches to quantifying DTI data have involved comparing the total number of abnormal values throughout the brain compared to a control group (as opposed to using focal region of interest analyses). Wäljas and colleagues [26] reported that at 3 weeks post-injury, civilian mTBI patients have significantly greater low FA values compared to healthy controls. Panenka *et al.* [14] reported that at approximately 6-8 weeks post-injury, civilian TBI patients (both complicated and uncomplicated cases), have a significant number of low FA values compared to a group of orthopedic trauma controls.

To complicate matters, there is evidence that traumatic stress is associated with differences in white matter integrity inferred from DTI. Several studies reveal that both veterans and civilians with PTS have white matter abnormalities of the cingulum bundle, superior longitudinal fasciculus, and corpus callosum [17-20, 27]. However, DTI findings in these same regions have also been associated with mTBI [28-30]. Thus, there is a need to explore if there are differences in the white matter integrity of these tracts in mTBI and PTS, compared to healthy controls.

The purpose of this study was to compare the clinical features and whole brain DTI results in three groups: active duty service members with a history of mTBI and current traumatic stress (mTBI/PTS), service members with traumatic stress only (PTS), and service members who deployed to the Middle East who do not have a history of mTBI or PTS (Controls). We hypothesized that the groups would differ in self-reported traumatic stress and postconcussive symptoms as follows: mTBI/PTS > PTS > Controls. We hypothesized a small difference in neuropsychological test results as follows: mTBI/PTS and PTS < Controls.

Regarding the DTI metrics, we hypothesized that when considering numerous regions of the brain simultaneously, the clinical groups would have a greater number of abnormal low FA values, as follows: mTBI/PTS > PTS > Controls. In light of the evidence showing that TBI is associated with diffusivity of brainstem nuclei [15, 31, 32], but not PTS; and reduced integrity of the cingulum bundle, superior longitudinal fasciculus, and corpus callosum has been observed in both PTS and TBI [17-20, 28-30], these tracts are specific regions of interest (ROIs) for this study. Therefore, we explored group differences in mean DTI values for FA and MD for specific commissural and association tracts, which included the superior longitudinal fasciculus, cingulum bundle, and corpus callosum because these tracts have been studied in association with PTS (and mTBI). We also examined projection tracts because, as aforementioned, they may be more vulnerable to damage associated with mTBI. These specific projection tracts included the cerebral peduncles and the corticospinal tract. We predicted that our results would show significant differences in mean DTI values in commissural and association tracts for both the PTS and PTS/mTBI groups compared to the control group, but only the PTS/mTBI group would have significant differences in the projection tracts.

## 2 MATERIALS AND METHODS

### 2.1 Participants

Participants were 76 active-duty U.S. Army soldiers recruited from Fort Rucker, AL and Fort Benning, GA, *via* flyers/posters, word of mouth, and clinician referrals. All eligible participants had a history of prior deployment(s) to the Middle East as part of Operations Iraqi Freedom and/or Operation Enduring Freedom. Prior to enrollment, candidates were pre-screened for MRI contraindication, TBI history, and symptoms to assess eligibility in a telephone interview. An independent study physician verified eligibility by screening soldiers’ electronic medical records for medical conditions that would exclude them from participation (*e.g.*, contraindications for MRI, failure to meet eligibility criteria). Soldiers that were eligible were consented. Testing took place at the Auburn University Magnetic Resonance Imaging Research Center, Auburn, AL. All participants passed standardized effort testing using the Test of Memory Malingering.

A deployment-exposed control group consisted of 30 healthy participants. Individuals were eligible to participate if they had no history of TBI which was also verified by their electronic medical records, they screened negative for posttraumatic stress (PTSD Checklist-5, PCL-5 < 20), and they had no psychiatric history or active diagnosis. Additionally, all participants had no contraindications for MRI.

A posttraumatic stress group (PTS) consisted of 16 participants who were eligible to participate if they screened positive for posttraumatic stress (PCL-5 ≥ 20), had no self-reported mTBI within the last five years or record of mTBI, no reporting or record of a past moderate-to-severe TBI, and no self-report or record of the diagnosis of substance dependency, mood and/or personality disorders.

A group of 28 participants with a medically documented history of mTBI and significant posttraumatic stress symptoms (PCL-5 ≥ 20) were recruited *via* clinician referrals. A diagnosis of mTBI required having been exposed to an injury event(s), and in the course of such an event(s), experienced an alteration in mental status (loss of memory, loss of consciousness, or seeing stars) for no greater than 30 minutes. This group is referred to as the PTS/mTBI group. Soldiers were eligible if they had a documented history of mTBI no earlier than three months and within the last five years, were symptomatic as assessed by the referring clinician and self-report (Neurobehavioral Symptom Inventory, NSI ≥ 24), had no record or self-reported history of moderate-to-severe TBI, and had no record or self-reported history of a diagnosis of substance dependency, mood and/or personality disorders.

The present study’s protocol was reviewed and approved by Institutional Review Boards from both Auburn University and the U.S. Army Medical Research and Materiel Command.

### 2.2 Psychological Health and Environment

Psychological health, environmental exposure, and health-related behaviors were assessed using a battery of common measures including the PTSD Checklist-5 (PCL-5) [33], Life Events Checklist [34], Childhood Environment scale [35], Zung Depression and Zung Anxiety Scales (ZDS/ZAS) [36, 37], Alcohol Use Disorders Identification Test (AUDIT) [38], Epworth Sleepiness Scale [39], and Neurobehavioral Symptom Inventory (NSI) [40]. In addition, participants were asked to report any prescribed medications they were taking.

### 2.3 Neurocognitive Assessment and Effort Testing

For neurocognitive assessment, we administered the Central Nervous System-Vital Signs^®^ (CNS-VS) [41] battery. The present study used five computerized CNS-VS subtests (verbal memory, symbol digit coding, Stroop test, continuous performance test, and the shifting attention test). The domain scores calculated were verbal memory (VM), complex attention (CA), reaction time (RT), processing speed (PS), cognitive flexibility (CF), and executive functioning (EF). All domain scores are presented as index scores, with a mean of 100 and standard deviation of 15. In addition, we also tested effort to improve the validity of our assessment data. To this end, we administered the Test of Memory Malingering [42], which consists of two learning trials and a retention trial that uses pictures of common, everyday objects (*e.g.*, chair, pencil). A cut-off score (*<*45 correct) was used to determine eligibility for participation in the study. All participants passed the TOMM on the first trial.

### 2.4 DTI Acquisition and Processing

Diffusion tensor imaging (DTI) (acquisition parameters: 25 slices acquired parallel to the AC-PC plane, voxel size = 1.8x1.8x3mm, TR/TE=3600/95, matrix=128x128, b=0, 1000, 30 directions) analyses were performed on 74 participants (controls, n = 30; PTS, n = 16; PTS/mTBI, n = 28). Diffusion weighted images were acquired as part of a sequence of structural and functional scans. In addition to the diffusion weighted image acquisition for the current study, participants also completed functional MRI (fMRI) scans during either an emotion regulation task (acquisition parameters: T2* weighted multiband EPI sequence, voxel size= 3.5×3.5×5 mm^3^, TR/TE=600/30, multiband-factor=2, with 680 volumes per run and 4 runs per subject) or fear conditioning task (acquisition parameters: T2* weighted EPI sequence, voxel size= 2×2×5 mm^3^, TR/TE=2000/35, with 450 volumes per run and 2 runs per subject), a high resolution anatomical MPRAGE scan (acquisition parameters: T1 weighted sequence, voxel size= 1×1×1 mm^3^, TR/TE=1900/2.5), and a resting state fMRI scan (acquisition parameters: T2* weighted multiband EPI sequence, voxel size= 3×3×4 mm^3^, TR/TE=600/30, multiband-factor=2, with 1000 volumes per subject). Diffusion weighted data were preprocessed and eddy corrected, after which the diffusion tensor model was fit to the data using FSL’s Diffusion Toolbox (FDT) DTIFIT program [43]. Voxelwise statistical analysis of the FA data was carried out using TBSS (Tract-Based Spatial Statistics) [44], part of FSL [45]. First, FA images were created by fitting a tensor model to the raw diffusion data using the FDT mentioned above, and then brain-extracted using BET [46]. All subjects' FA data were then aligned into a common space using the nonlinear registration tool FNIRT [47], which uses a b-spline representation of the registration warp field [48]. Next, the mean FA image was created and thinned to create a mean FA skeleton, which represents the centers of all tracts common to the group. Each subject's aligned FA data was then projected onto this skeleton and the resulting data fed into voxelwise cross-subject statistics for group comparisons, controlling for age and gender, using RANDOMISE.

DTI FA and MD values were computed on 38 tract-based regions of interest (ROIs) rendered from the Johns Hopkins University (JHU) DTI-based white matter atlas [49-51]. These included the singular*, and left and right hemisphere ROIs of the following tracts: the anterior limb of internal capsule, posterior limb of internal capsule, retrolenticular part of internal capsule, anterior corona radiata, body of corpus callosum*, cingulum-hippocampus, cingulum-cingulate gyrus, cerebral peduncle, middle cerebral peduncle*, inferior cerebral peduncle, external capsule, fornix*, fornix stria terminalis, genu-corpus callosum*, corticospinal tract, posterior corona radiata, posterior thalamic radiation, sagittal stratum, superior longitudinal fasciculus, splenium-corpus callosum*, superior corona radiata, superior fronto-occcipital fasciculus, tapetum, and uncinate fasciculus.

The number of ROIs with FA and MD values that fell below/above a specified cut score for each participant was calculated. The cut scores were identified by calculating the means and standard deviations (SD) for FA and MD values at each of the 38 ROIs from the healthy control group’s data. FA values that were <2 SDs below the mean, and MD values >2 SDs above the mean were classified as abnormal scores (*i.e.*, having compromised white matter integrity).

### 2.5 Statistical Analyses

Data analysis was conducted using IBM Statistical Package for the Social Sciences (IBM SPSS 19). Kruskal-Wallis test was used to compare differences between the groups for psychological health scores, neurocognitive scores, the number of abnormal ROIs, and mean diffusivity values for FA and MD. A priori comparisons of mean DTI values for specific ROIs included: (i) *projection tracts*: cerebral peduncle, middle cerebral peduncle, inferior cerebral peduncle, and corticospinal tract; (ii) *commissural tracts*: cingulum bundle (cingulum-cingulate gyrus and cingulum-hippocampus) and corpus callosum (body of the corpus callosum and genu); and (iii) *association tracts*: superior longitudinal fasciculus. Benjamini-Hochberg corrections were applied to control for false discovery rate (FDR) during primary group analyses: 9 comparisons for demographic and psychological health variables; 6 comparisons for neurocognitive measures; 10 comparisons for projection tracts; 10 comparisons for commissure tracts; and 4 comparisons for association tracts. Post hoc Dunnett’s C corrections were used in pairwise comparisons to control for familywise error rate (FWE). Other exploratory post hoc analyses used either Chi-square test (χ^2^) or Mann-Whitney U. Cohen’s *d* was used for assessing effect sizes (small = 0.2, medium = 0.5, large = 0.8).

## 3 RESULTS

### 3.1 Demographics

Descriptive statistics and group comparisons for demographic, neurocognitive, and psychological health measures between groups are presented in Table (**1**). There was a difference in gender in that the PTS/mTBI group’s participants were all men compared to 73% men in the control group. In addition, the PTS/mTBI group was less educated and had higher levels of prior traumatic events compared to controls, but no difference in their childhood environment or age. For the PTS group, only the level of traumatic life events was significantly higher compared to controls. There were no significant differences between the PTS and the PTS/mTBI groups amongst demographic variables.

### 3.2 Neurocognition, Psychological Health, Environment, and Medications

As observed in Table (**1**), there were significant group differences on a number of psychological health and select neurocognitive domain scores. Effect sizes for significant differences ranged from medium to very large (*d* = 0.77 to 5.03). Overall, the PTS/mTBI group had greater levels of traumatic stress, anxiety, depression, and post-concussive symptoms than both the PTS and control groups, and higher levels of alcohol consumption than the control group. The PTS group had greater levels of traumatic stress, depression, and postconcussive symptoms than the control group. There were no significant differences between the PTS group and control group on neurocognitive testing. As shown in Table (**2**), the PTS/mTBI group reported using a greater number of prescription medications than both the PTS and control groups.

### 3.3 Frequencies of Abnormal DTI ROIs

Comparing the three groups using Kruskal-Wallis test, there were no omnibus differences in the number of abnormal FA or MD DTI scores (Table **3**).

For exploratory purposes, cumulative percentages of participants with abnormal FA and MD scores for the PTS/mTBI, PTS, and control groups, and the differences between these groups, are presented in Table (**4**). There were no statistically significant differences in the rates of abnormal FA or MD scores between any of the three groups. Interestingly, the table suggests one control had a number of abnormal FA and MD scores. Only when the cumulative percent in the control group (3.3%) was removed did there emerge a significant difference, specifically with the PTS/mTBI group having a greater number of participants with both five (17.9%; χ^2^ = 5.8, *p* = .016) and six (14.3%; χ^2^ = 4.5, *p* = .033) abnormal FA scores compared to controls (0.0%).

### 3.4 Commissure, Association, and Projection Tracts

Group comparisons using Kruskal-Wallis test (Benjamini-Hochberg corrected) of the 4 projection tracts ROIs revealed omnibus significant differences in MD (*p* = 0.25) values for the right corticospinal tract with greater diffusivity in the PTS/mTBI group compared to controls. However, this was no longer significant after correcting for potential FDR. There were no significant group differences in mean FA or MD values of the specific commissural and association ROIs.

## 4 DISCUSSION

The findings of the current study revealed that soldiers with PTS and mTBI had significantly greater traumatic stress, depression, anxiety, and post-concussive symptoms and performed worse on neurocognitive testing than those with PTS alone and controls. In contrast, the we did not find strong evidence of compromised white matter integrity in either the PTS or PTS/mTBI groups compared to controls. This was demonstrated in using several different analytic approaches. The first compared the number of abnormal DTI values for FA and MD values. Only after removing data from the controls that may have been associated with an outlier was there a significant difference in the PTS/mTBI group showing a greater number of participants with five and six abnormal FA scores. Next, we explored differences in mean DTI values across a number of different white matter tracts. While we initially discovered significant differences in the cortical spinal tract for the PTS/mTBI group, when corrections were applied to control for false discovery rate, this difference was no longer significant.

Our imaging findings partially support those reported by both Panenka *et al.* [14] and Wäljas *et al.* [26]. This was unexpected since we predicted that the PTS/mTBI group that would have a greater number of individuals with abnormal DTI scores compared to controls, especially since these participants were the most severe in terms of clinically relevant symptoms and neurocognitive scores. Further, we expected to observe mean group differences over several neuroanatomical locations that would reflect qualitative differences between the clinical groups; namely a history of mTBI(s). However, there were considerable differences in our sample of participants compared to Panenka *et al.* and Wäljas *et al.* in that they sampled from civilian patients with more recent injuries (6-8 weeks and 3-weeks post-injury, respectively). In contrast, our participants consisted of active-duty soldiers who sustained an mTBI between 12 months and 5 years. Another difference was the prevalence of PTS in our population.

An alternative explanation for the lack of robust imaging findings between the clinical groups is that the PTS/mTBI group’s clinical presentation might be driven by the severity of their PTS and not necessarily pathoanatomical changes from mTBI. The group did report significantly greater symptoms in traumatic stress, depression, anxiety, and post-concussive symptoms than the PTS alone group. Yet, if this was the case, one might expect to observe abnormal DTI values based on evidence that prolonged stress is associated with reduced white matter integrity [17-20, 27].

The initial, uncorrected, finding of a difference in mean MD values of the cortical spinal tract of the PTS/mTBI group is supported by evidence suggesting brainstem nuclei are vulnerable to the biomechanics of mTBI more than other regions with greater neuronal density [15, 31, 32]. However, these differences were no longer significant after controlling for multiple comparisons. Therefore, abnormalities in the cortical spinal tract in this highly comorbid sample of soldiers may not be a reliable biomarker.

The superior longitudinal fasciculus (SLF) is a major association tract that subserves fronto-parietal integration and has been implicated in both brain injury [52] and depression [53]. This has been observed in depressed OEF/OIF veterans with a history of mTBI [54]. In addition, there is evidence of depression impacting integrity of the cingulum-hippocampal tract (CH) in OEF/OIF veterans with PTSD and co-morbid mTBI [55]. Both of our clinical groups had significantly elevated levels of depression, with the PTS/mTBI group being the most severe. However, there were no significant group differences in diffusivity of the SLF tract.

There were several limitations to the current study. First, although both of the clinical target groups had PTS, these symptoms were significantly greater in the PTS/mTBI group compared to both the controls and PTS group. Future efforts should be made to control for PTS symptom severity by recruiting service members with mTBI alone (*i.e.*, no PTS). Second, differences in reported prescription medication varied between the groups, with the co-morbid group having the highest percentage of medicated participants. Future studies should focus on medication-naïve participants in order to get a better understanding of the impact of PTS and mTBI. Third, we were not able to determine the frequency or temporal information of mTBIs the comorbid group sustained during the specified period of five years (> three months) at the time of participation in the study or across any of the participants’ lifetime. Fourth, only after removing data from the controls that suggested a potential outlier did we find significant differences between controls and the PTS/mTBI group, albeit small, in the cumulative percentage of participants with abnormal FA scores. However, a degree of experimenter bias is introduced in that it is likely phenotypic morphologic differences contribute to variance in the general population as well as our clinical populations. Finally, although there were some statistically significant imaging findings, the effect sizes were small and the sample size modest, and when corrected for multiple comparisons were no longer significant; hence, illustrating the need for replication in future studies with larger sample sizes.

## CONCLUSION

In conclusion, our findings do not provide strong evidence of compromised white matter integrity between our clinical groups compared to controls using several analytic approaches. In contrast, our groups were best categorized by robust differences in clinical symptoms and neurocognitive scores. As such, our findings suggest that psychological health conditions rather than pathoanatomical changes may be contributing to symptoms presented by soldiers with comorbid PTS and mTBI.

## Figures and Tables

**Table 1:** Group differences in demographic, psychological health, and neurocognitive metrics.

**Demographic**	**Controls**	**PTS**	**PTS/mTBI**
Gender, Men/Women^a^	22/30	15/16	28/28*
F			
%	73%	94%	100%
Age			
M	30.6	32.5	32
SD	5.7	7.1	5.4
d		0.3	0.3
Education			
M	15.4	14.5	13.9*
SD	2.1	1.9	1.5
d		0.4	0.8
Life Events			
M	27.0	44.8*	42.4*
SD	12.0	9.8	13.1
d		1.7	1.6
Childhood Environment			
M	57.4	53.4	52.6
SD	10.9	9.8	13.1
d		0.4	0.4
**Psychological Health**			
Traumatic Stress			
M	3.6	32.5*	51.7**
SD	4.7	10.3	14.8
d		4.4	5.0
Depression			
M	32.0	40.4*	52.6**
SD	7.0	11.3	9.8
d		1.0	2.5
Anxiety			
M	30.4	38.4*	51.1**
SD	7.0	11.9	9.8
d		0.9	2.5
Postconcussive			
M	6.6	24.7*	44.5**
SD	6.0	15.7	16
d		2.0	3.5
Alcohol Use			
M	4.6	7.2	9.1*
SD	3.6	6.7	8.1
d		0.6	0.8
**Neurocognition** (CNS-Vital Signs)			
Reaction Time			
M	99.9	96.0	83.8
SD	18.6	13.2	29.5
d		0.2	0.7
Complex Attention			
M	94.3	88.0	74.7*
SD	19.1	17.9	22.9
d		0.3	0.9
Cognitive Flexibility			
M	101.3	100.6	80.6**
SD	18.2	13.1	22.5
d		0.0	1.0
Processing Speed			
M	105.4	101.9	91.3*
SD	22.0	13.2	15.3
d		0.2	0.8
Executive Function			
M	103.9	103	86.6**
SD	16.7	11.7	21.3
d		0.1	0.9
Verbal Memory			
M	97.8	100.5	85.9
SD	21.4	19.9	23.9
d		0.1	0.5

Note: Controls (n=30), PTS (n=16), PTS/mTBI (n=28). Kruskal-Wallis test (Benjamini-Hochberg Corrected) with post hoc comparisons (Mann-Whitney test). ^a^ *χ^2^* test. *Significant compared to the control group; **Significant between both control and PTS groups; F = Frequency; M = Mean; SD = Standard Deviation; d = Cohen’s d

**Table 2:** Frequencies of psychoactive medication use by group.

Medication, (%)		**Controls**	**PTS**	**PTS/mTBI**
Antidepressants		3 (1%)	3 (19%)	10 (36%)
	^a^two or more	0	1 (6%)	4 (14%)
Antipsychotics		1 (3%)	0	2 (7%)
Benzodiazepines		0	2 (13%)	5 (18%)
Mood stabilizers		0	0	0
Stimulant		0	1 (6%)	1 (4%)
Hypnotic (Nonbenzodiazepine)		0	0	4 (14%)
Anxiolytic		0	1 (6%)	1 (4%)
Opioid analgesic		1 (3%)	0	2 (2%)

^a^Two or more types of antidepressants

**Table 3:** Descriptive statistics of the number of abnormal DTI scores across 38 ROIs by group.

	1. *PTS/mTBI*		2. *PTS*		3. *Controls*		Effect Sizes		
							1 v 2	1 v 3	2 v 3
	M	SD	M	SD	M	SD			
Number of low FA scores	2.4	5.8	2.1	2.5	1.4	2.3	.10	.28	.30
Number of high MD scores	2.4	2.7	2.5	3.6	1.6	2.6	.05	.31	.30

Note: Kruskal-Wallis Test across 3 groups: FA (*p* =.383); MD (*p* = .314). FA=fractional anisotropy, MD=mean diffusivity; M = Mean; SD = Standard Deviation. *p < .05; (Pairwise comparisons; Dunnett’s C) Effect Sizes = Cohen’s *d*

**Table 4:** Cumulative percentage of the number of abnormal FA and MD scores across 38 ROIs.

	**FA**						**MD**					
	1. PTS/mTBI	2. PTS	3. Controls	% Diff			1. PTS/mTBI	2. PTS	3. Controls	% Diff		
				1 v 2	1 v 3	2 v 3				1 v 2	1 v 3	2 v 3
	%	%	%	%	%	%	%	%	%	%	%	%
24 or more	0	6.3	0	-6.3	0.0	6.3	0	0	0	0.0	0.0	0.0
~	-	-	-	-	-	-	-	-	-	-	-	-
14 or more	0	6.3	0	-6.3	0.0	6.3	3.6	0	0	3.6	3.6	0.0
13 or more	0	6.3	0	-6.3	0.0	6.3	3.6	0	0	3.6	3.6	0.0
12 or more	0	6.3	0	-6.3	0.0	6.3	3.6	0	3.3	3.6	0.3	-3.3
11 or more	0	6.3	3.3	-6.3	-3.3	3.0	7.1	0	3.3	7.1	3.8	-3.3
10 or more	0	6.3	3.3	-6.3	-3.3	3.0	7.1	0	3.3	7.1	3.8	-3.3
9 or more	3.6	6.3	3.3	-2.7	0.3	3.0	7.1	6.3	3.3	0.8	3.8	3.0
8 or more	3.6	6.3	3.3	-2.7	0.3	3.0	10.7	12.5	3.3	-1.8	7.4	9.2
7 or more	7.1	6.3	3.3	0.8	3.8	3.0	10.7	12.5	3.3	-1.8	7.4	9.2
6 or more	14.3	6.3	3.3	8.0	11.0*	3.0	14.3	12.5	3.3	1.8	11.0	9.2
5 or more	17.9	6.3	3.3	11.6	14.6*	3.0	17.9	12.5	16.7	5.4	1.2	-4.2
4 or more	25.0	6.3	10.0	18.7	15.0	-3.7	25.0	18.8	20.0	6.2	5.0	-1.2
3 or more	28.6	18.8	23.3	9.8	5.3	-4.5	39.3	37.5	20.0	1.8	19.3	17.5
2 or more	50.0	25.0	33.3	25.0	16.7	-8.3	46.4	56.3	26.7	-9.9	19.7	29.6
1 or more	57.1	68.8	50.0	-11.7	7.1	18.8	57.1	68.8	50.0	-11.7	7.1	18.8
0 scores	100	100	100	0.0	0.0	0.0	100	100	100	0.0	0.0	0.0

Note: PTS/mTBI (n=28), PTS (n=16), DEC (n=30). Abnormal FA < 2SDs compared to the control group mean; Abnormal MD > 2SDs compared to the control group mean. FA=fractional anisotropy and MD=mean diffusivity. *Significant (χ^2^, *p* < .05) after removing the cumulative percentage in the control group (3.3%) which might reflect a potential outlier.
